# Draft Genome Sequence of *Rhodosporidium toruloides* CECT1137, an Oleaginous Yeast of Biotechnological Interest

**DOI:** 10.1128/genomeA.00641-14

**Published:** 2014-07-10

**Authors:** Nicolas Morin, Xavier Calcas, Hugo Devillers, Pascal Durrens, David James Sherman, Jean-Marc Nicaud, Cécile Neuvéglise

**Affiliations:** a INRA, UMR1319 Micalis, Jouy-en-Josas, France; b AgroParisTech, UMR Micalis, Jouy-en-Josas, France; c INRIA-LaBRI-Université de Bordeaux, Magnome, Bordeaux, France

## Abstract

We report the sequencing of the basidiomycetous yeast *Rhodosporidium toruloides* CECT1137. The current assembly comprises 62 scaffolds, for a total size of ca. 20.45 Mbp and a G+C content of ca. 61.9%. The genome annotation predicts 8,206 putative protein-coding genes.

## GENOME ANNOUNCEMENT

Large-scale production of biodiesel requires considerable amounts of fatty acids (FAs). The resulting demand for FAs has made oleaginous yeast species a target of choice as an alternative source of oils and fats (1). The genomic data available for such species have increased over the last few years but still remain scarce. To increase the current knowledge of oleaginous yeast species, we report the sequencing of the basidiomycetous yeast *Rhodosporidium toruloides* CECT1137. This strain was originally deposed in the Natural Collection of Yeast Cultures (NCYC) (United Kingdom) in 1925 by Antoine Guillermond (strain NCYC162) under the name “Levure de rose.” It was transferred in 1984 to the Spanish Type Culture Collection (CECT) (Spain), where it received the CECT1137 accession number. This strain was originally described as *Rhodotorula glutinis* var. *glutinis* based on physiological characteristics. However, our sequencing data reinstated it as a member of the species *R. toruloides*.

The CECT1137 genome was sequenced by Eurofins Scientific (France), using Roche 454 GS FLX Titanium, on a single-read library and an 8-kb mate-pair library. A complementary run of Illumina HiSeq 2000 was performed on a cDNA library using Illumina TruSeq RNA to improve the predictions of the gene models and their numerous introns. Several assembly runs were performed using a combination of MIRA (2) and Allpaths-LG (3). Smoothing and scaffolding were performed using PILON (version 1.7; The Broad Institute [http://www.broadinstitute.org/software/pilon/]) and SSPACE-BASIC-2.0 (4). Genes were predicted using a combination of tools, including Augustus (5), GeneMark (6), EST2Genome (7), BLASTp, and PSItblastn (8). The recently published genome of the closely related strain NP11 was integrated in the annotation pipeline as an additional support for prediction (9). The cDNA reads were mapped on the genome using Tophat2 (10). Exon-exon junctions were extracted from the alignments using a combination of in-house tools developed in BioPerl (11). Splicing events and structural annotation were manually validated using the Artemis software (12). The tRNA genes were identified using tRNAscan-SE (13).

The current draft comprises 62 scaffolds, for a total size of 20,445,260 bp and a G+C content of ca. 61.9%. Overall, 8,206 putative protein-coding genes have been identified, 212 of which harbor introns alternatively spliced within the coding sequences and/or the untranslated regions (UTR). An additional 145 genes have been annotated as dubious models or pseudogenes, with frameshifts, stops in translation, or dubious starts or stops. The genome contains 149 tRNAs and 249 miscellaneous RNAs. Whenever possible, a functional annotation was proposed, based on a combination of BLASTp (8), EMBOSS Needle (7), and InterProScan (14) against subsets of genes extracted from UniProt (15), with priority given to experimentally validated data from yeasts and fungal species. A total of 8,294 proteins were predicted (including 88 splicing variants), among which 7,080 showed at least 20% sequence similarity on 70% of an alignment with at least one gene from the Swiss-Prot/TrEMBL subsets.

Further comparison of the genome of CECT1137 against other yeast species will bring additional insights on the genomic properties of oleaginicity, providing potential targets for future biotechnological applications.

### Nucleotide sequence accession numbers.

This whole-genome shotgun project has been deposited at the European Nucleotide Archive under the accession no. LK052936 to LK052997.

## References

[1] Beopoulos A Nicaud JM Gaillardin C 2011 An overview of lipid metabolism in yeasts and its impact on biotechnological processes. Appl. Microbiol. Biotechnol. 90:1193–1206. 10.1007/s00253-011-3212-8 21452033

[2] Chevreux B 2005 MIRA: an automated genome and EST assembler. Ph.D. thesis The Ruprecht-Karls-University, Heidelberg, Germany

[3] Butler J MacCallum I Kleber M Shlyakhter IA Belmonte MK Lander ES Nusbaum C Jaffe DB 2008 ALLPATHS: *de novo* assembly of whole-genome shotgun microreads. Genome Res. 18:810–820. 10.1101/gr.7337908 18340039 PMC2336810

[4] Boetzer M Henkel CV Jansen HJ Butler D Pirovano W 2011 Scaffolding pre-assembled contigs using SSPACE. Bioinformatics 27:578–579. 10.1093/bioinformatics/btq683 21149342

[5] Stanke M Steinkamp R Waack S Morgenstern B 2004 Augustus: a Web server for gene finding in eukaryotes. Nucleic Acids Res. 32:W309–W312. 10.1093/nar/gkh379 15215400 PMC441517

[6] Besemer J Borodovsky M 2005 GeneMark: Web software for gene finding in prokaryotes, eukaryotes and viruses. Nucleic Acids Res. 33:W451–W454. 10.1093/nar/gki487 15980510 PMC1160247

[7] Rice P Longden I Bleasby A 2000 EMBOSS: the European Molecular Biology Open Software Suite. Trends Genet. 16:276–277. 10.1016/S0168-9525(00)02024-2 10827456

[8] Altschul SF Gish W Miller W Myers EW Lipman DJ 1990 Basic Local Alignment Search Tool. J. Mol. Biol. 215:403–410. 10.1016/S0022-2836(05)80360-2 2231712

[9] Zhu Z Zhang S Liu H Shen H Lin X Yang F Zhou YJ Jin G Ye M Zou H Zhao ZK Zhao ZK 2012 A multi-omic map of the lipid-producing yeast *Rhodosporidium toruloides*. Nat. Commun 3:1112. 10.1038/ncomms2112 23047670 PMC3493640

[10] Kim D Pertea G Trapnell C Pimentel H Kelley R Salzberg SL 2013 TopHat2: accurate alignment of transcriptomes in the presence of insertions, deletions and gene fusions. Genome Biol. 14:R36. 10.1186/gb-2013-14-4-r36 23618408 PMC4053844

[11] Stajich JE Block D Boulez K Brenner SE Chervitz SA Dagdigian C Fuellen G Gilbert JG Korf I Lapp H Lehväslaiho H Matsalla C Mungall CJ Osborne BI Pocock MR Schattner P Senger M Stein LD Stupka E Wilkinson MD Birney E 2002 The Bioperl toolkit: Perl modules for the life sciences. Genome Res. 12:1611–1618. 10.1101/gr.361602 12368254 PMC187536

[12] Rutherford K Parkhill J Crook J Horsnell T Rice P Rajandream MA Barrell B 2000 Artemis: sequence visualization and annotation. Bioinformatics 16:944–945. 10.1093/bioinformatics/16.10.944 11120685

[13] Lowe TM Eddy SR 1997 tRNAscan-SE: a program for improved detection of transfer RNA genes in genomic sequence. Nucleic Acids Res. 25:955–964. 10.1093/nar/25.5.0955 9023104 PMC146525

[14] Zdobnov EM Apweiler R 2001 InterProScan—an integration platform for the signature-recognition methods in InterPro. Bioinformatics 17:847–848. 10.1093/bioinformatics/17.9.847 11590104

[15] Uniprot Consortium. 2014 Activities at the Universal Protein Resource (UniProt). Nucleic Acids Res. 42:D191–D198. 10.1093/nar/gkt1140 24253303 PMC3965022

